# The Long-Term Differentiation of Embryonic Stem Cells into Cardiomyocytes: An Indirect Co-Culture Model

**DOI:** 10.1371/journal.pone.0055233

**Published:** 2013-01-28

**Authors:** Dong-Bo Ou, Di Zeng, Yan Jin, Xiong-Tao Liu, Ji-Wei Teng, Wan-Gang Guo, Hong-Tao Wang, Fei-Fei Su, Yong He, Qiang-Sun Zheng

**Affiliations:** 1 Department of Cardiology, Tangdu Hospital, Fourth Military Medical University, Xi’an, China; 2 Research and Development Center for Tissue Engineering, Fourth Military Medical University, Xi’an, China; University of Udine, Italy

## Abstract

**Background:**

Embryonic Stem Cells (ESCs) can differentiate into cardiomyocytes (CMs) *in vitro* but the differentiation level from ESCs is low. Here we describe a simple co-culture model by commercially available Millicell™ hanging cell culture inserts to control the long-term differentiation of ESCs into CMs.

**Methodology/Principal Findings:**

Mouse ESCs were cultured in hanging drops to form embryoid bodies (EBs) and treated with 0.1 mmol/L ascorbic acid to induce the differentiation of ESCs into CMs. In the indirect co-culture system, EBs were co-cultured with epidermal keratinocytes (EKs) or neonatal CMs (NCMs) by the hanging cell culture inserts (PET membranes with 1 µm pores). The molecular expressions and functional properties of ESC-derived CMs in prolonged culture course were evaluated. During time course of ESC differentiation, the percentages of EBs with contracting areas in NCMs co-culture were significantly higher than that without co-culture or in EKs co-culture. The functional maintenance of ESC-derived CMs were more prominent in NCMs co-culture model.

**Conclusions/Significance:**

These results indicate that NCMs co-culture promote ESC differentiation and has a further effect on cell growth and differentiation. We assume that the improvement of the differentiating efficiency of ESCs into CMs in the co-culture system do not result from the effect of co-culture directly on cell differentiation, but rather by signaling effects that influence the cells in proliferation and long-term function maintenance.

## Introduction

Embryonic stem cells (ESCs) derived from the inner cell mass of preimplantation mammalian embryos can be propagated in undifferentiated state maintaining their pluripotency to form various kinds of adult tissue cells [1]. Under appropriate conditions, ESCs can form embryoid bodies (EBs) and subsequently differentiate into cardiomyocytes (CMs) that retain the function of excitability and spontaneous contractions [2], [3]. The availability of ESCs and their successful differentiation into genuine cardiac cells have enabled researchers to gain novel insights into the early development of the heart as well as to pursue the revolutionary paradigm of heart regeneration.

Many factors have already been shown to be involved in the cardiomyocyte (CM) differentiation from ESCs, including 5-azacytidine, retinoic Acid (RA), ascorbic acid, endothelin, oxytocin, hepatocyte growth factor (HGF), transforming growth factor beta1 (TGF-β1), activin–A, and bone morphogenic protein (BMP)-2/4, and so on [4], [5], [6], [7], [8], [9], [10], [11], [12]. However, in many cases, simple differentiating factor fails to maintain the lineage-specific differentiation from ESCs. The majority of ESC-derived CMs (ESCMs) lost their automaticity and ceased spontaneous beating during long-term culture [13]. Although CMs can be efficiently derived from ESCs, the long-term maintenance of structural and functional properties of these ESCMs needs more research.

It is reported that the CM differentiation of ESCs requires a paracrine pathway in the heart [14]. When transplanted into infarcted mouse hearts, the ESC-derived cardiac progenitor cells can differentiate into cross-striated CMs forming gap junctions with the host cells [15]. This indicates an important role of microenvironment in facilitating CM differentiation of ESCs. Cell microenvironment created by co-culture with defined cells, mimic *in vivo* physiological environment, is considered to be important in directing the site-specific differentiation of ESCs. For example, when ESCs are co-cultured with visceral-endoderm-like (END-2) cells, there are 90% ESC-derived CMs similar to fetal ventricular cells [16]. When ESCs were cultured in conditioned medium from END-2 cells, the cardiogenic differentiation of ESCs can be readily enhanced [17]. Similarly, when conditioned medium from mouse embryo fibroblasts is used, the homogeneity of beating EBs can be significantly improved [18]. Although co-culture with defined cells are proved effective for CM differentiation, detailed characterization of this system on long-term differentiation of ESCs is generally lacking.

Previously, we investigated the effect of *in vitro* cardiac microenvironment on the development of EB growth and CM differentiation and had established a novel ESC differentiation model that can reproduce the early process of cardiovascular development [19], [20]. Nevertheless, the long-term development and functional maintenance of ESCMs have not yet been studied. Here, based on previous ascorbic acid-induced CM differentiation from ESCs, we sought to determine the role of local microenvironments created by co-culture with neonatal CMs (NCMs) in the EB development and CM differentiation that focuses on homogenous differentiation and long-term functional maintenance of the ESCMs.

## Results

### The CM Differentiation of ESCs in the Indirect Co-culture Model

**Figure 1:**
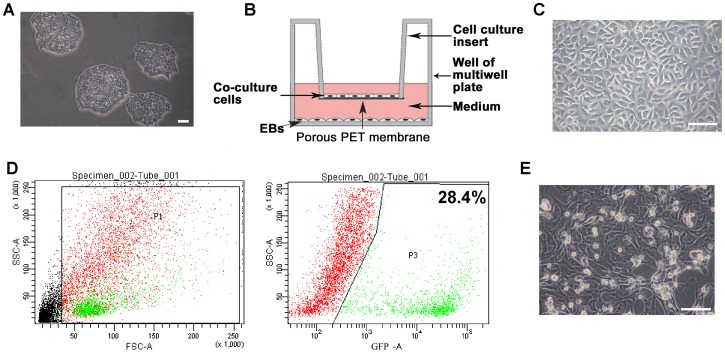
The indirect co-culture model. A, Morphology of undifferentiated ESC colonies cultured without feeder layer. **B,** Scheme of the indirect co-culture system: a facile cell expansion and stem cell differentiation system with continuous medium conditioning while preventing mixing of stem cells and co-culture cells by hanging culture inserts. Two cell populations are co-cultured in different compartments (insert and well) but can communicate via paracrine signaling through the pores of the membrane. **C,** The morphology of EKs. **D,** Reporter-based fluorescence-activated cell sorting (FACS) for purifying GFP^+^ cardiomyocytes isolated from neonatal αMHC-GFP transgenic mice. **E,** The morphology of the purified cardiomyocytes. Scale bars = 100 µm.

Undifferentiated ESCs were cultured on gelatin-coated dishes without feeder layer in the mentioned ESC medium (Figure 1A). In the indirect co-culture model, the co-culture cells were seeded on 6- or 12- well hanging cell culture inserts to prevent direct contact with the subnatant EBs (Figure 1B). EKs, obtained from the skin of newborn (2–3-day old) mice, were used as negative co-culture cells to better assess the differentiating potential of NCMs (Figure 1C). To ensure the purity of isolated NCMs population, we generated αMHC promoter driven eGFP-Rex-Neomycin transgenic mice (αMHC-GFP), in which only mature cardiomyocytes but not other cell types expressed the green fluorescent protein (GFP). The isolated NCMs were quantitatively purified through reporter-based fluorescence-activated cell sorting (FASC) (Figure 1D, E).

**Figure 2:**
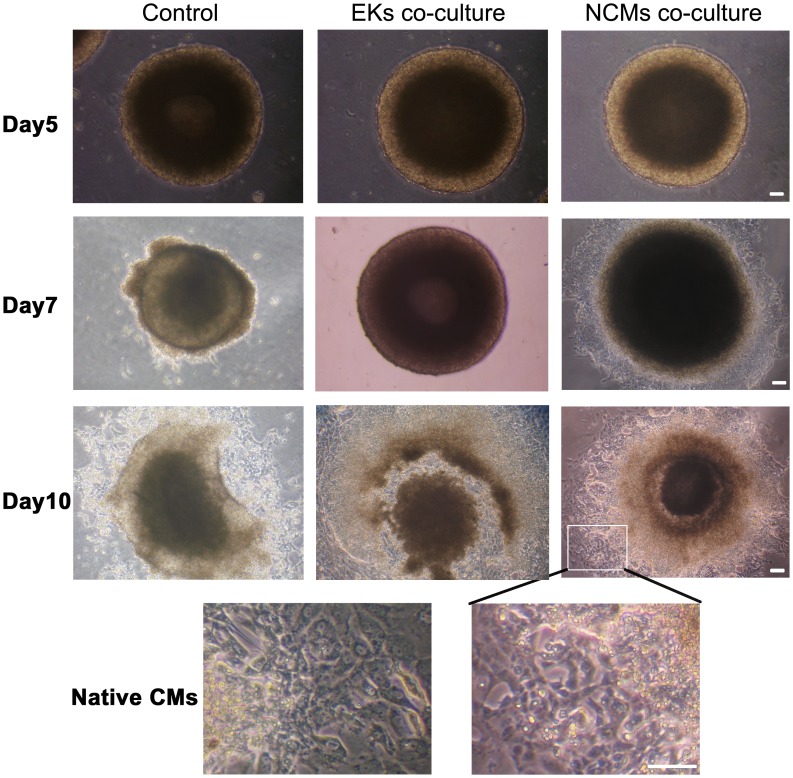
CM differentiation from ESCs in the indirect co-culture model. Morphology of 5-, 7- and 10-day-old EBs during ESCs differentiation. Hanging inserts were removed when photographed. In NCMs co-culture group, the EB outgrowths had a similar morphology to native CMs at day 10 of differentiation. Scale bars = 100 µm.

Under differentiation conditions, ESCs consistently aggregated and formed EBs. Figure 2 show EBs photographed from 5 to 10 days after initiation of cellular aggregation of the ESCs. Initially, EBs were formed by hanging drop culture and largely composed of densely packed ESCs. After suspension culture for 4 days, the EBs adhered to plates and the center of the bodies became cavitated. The rhythmically contracting areas consisted of 10 to 200 CMs began to appear in EBs, suggesting the occurrence of CM differentiation of ESCs. Beating EBs were first observed approximately at day 7 of differentiation. Starting on day 10 of differentiation, areas of rhythmically contracting cells in solid aggregates became evident, with more similar morphology to native CMs in NCMs co-culture (Figure 2).

### Co-culture with NCMs Improve the Differentiation Efficiency

**Figure 3:**
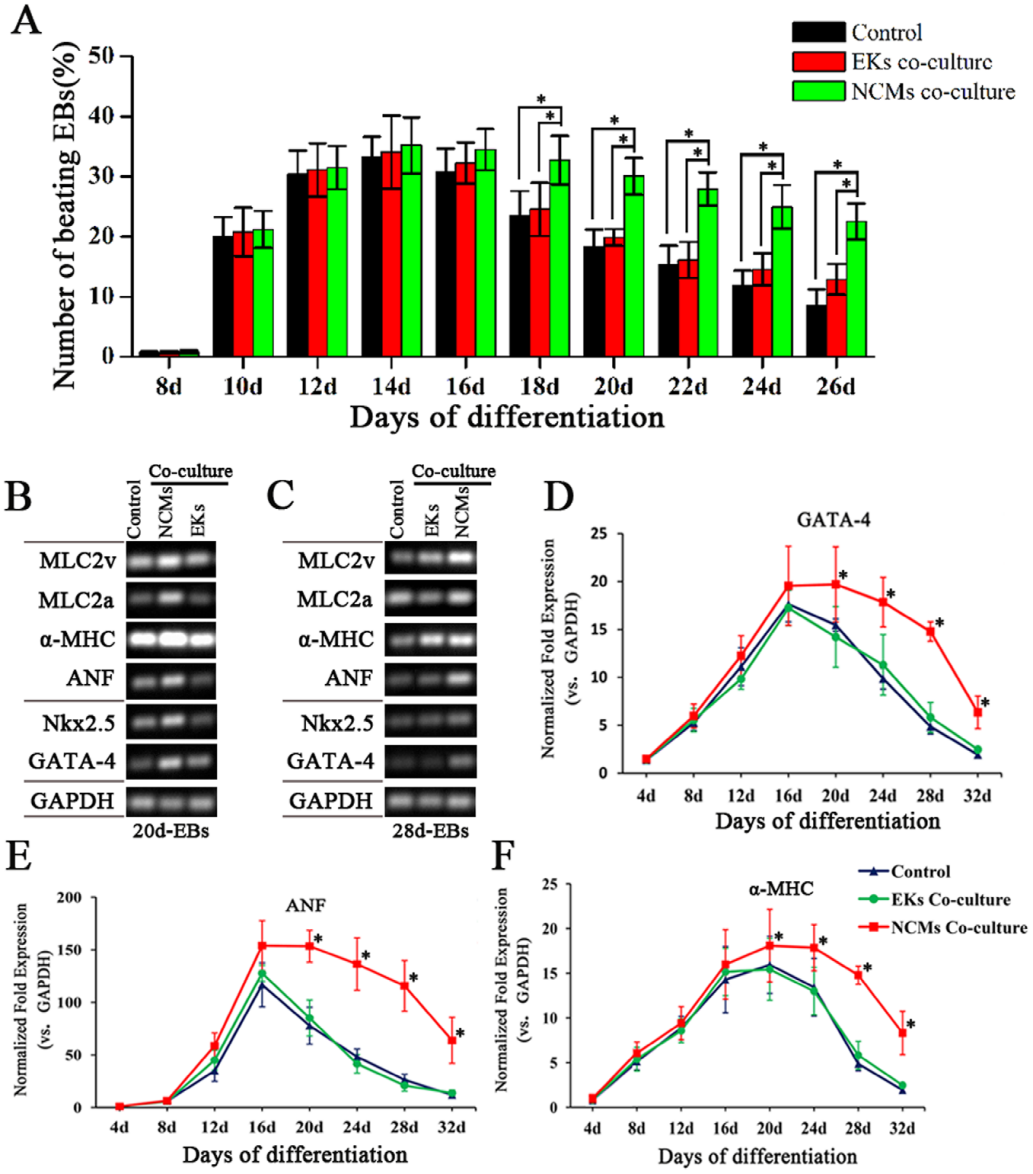
Effect of NCMs co-culture on the differentiation efficiency of ESCs. **A,** Time course quantification of spontaneous beating activity of differentiated cardiomyocytes was expressed as the percentage of beating EBs. **B and C,** semi-quantitative RT-PCR analysis on cardiac-specific markers (GATA-4, Nkx2.5, ANF, α-MHC, and MLC2a/2v) expression of 20- and 28-day-old EBs. **D, E and F,** Time course quantification of GATA-4, ANF and α-MHC mRNA expression by Real time-PCR. Expression levels of each gene were normalized to GAPDH.The fold change is expressed as mean±SEM (*n = *3–11). *: P<0.01.

During time course of ESC differentiation, the percentage of EBs with contracting areas in NCMs co-culture was significantly higher than that without co-culture or in EKs co-culture. NCMs co-culture did influence the CM differentiation rate of ESCs in intermediate-stage and late-stage (Figure 3A).

To verify the promoting effect of NCMs co-culture on CM differentiation of ESCs, the expression of cardiac marker genes were analyzed by semi-quantitative and real-time PCR. GATA-binding protein 4 (GATA-4) and NK2 transcription factor related locus 5 (Nkx2.5) were included as markers for cardiac mesoderm. α-myosin heavy chain (α-MHC), atrial natriuretic factor (ANF) and myosin light chain 2 atrial and ventricular transcripts (MLC2a, MLC2v) were included as markers for cardiomyocytes. Semi-quantitative RT-PCR analysis on 20- and 28-day-old EBs demonstrated that the expressions of above cardiac-specific markers were increased significantly with NCMs co-culture (Figure 3 B, C). Prolonged time course analysis with real time-PCR revealed that co-culture with NCMs could increase and maintain the expression of GATA-4, ANF, and α-MHC in a relatively sustained *manner* (Figure 3D,E,F). As early as day 4, GATA-4 expression was detected and significantly increased after day 20 in NCMs co-culture, compared to that of control group and EKs co-culture group (P<0.01). Similar to GATA-4, ANF and α-MHC were expressed at day 8 and their expressions were maintained in higher lever with NCMs co-culture after day 20 of differentiation (P<0.01).

**Figure 4:**
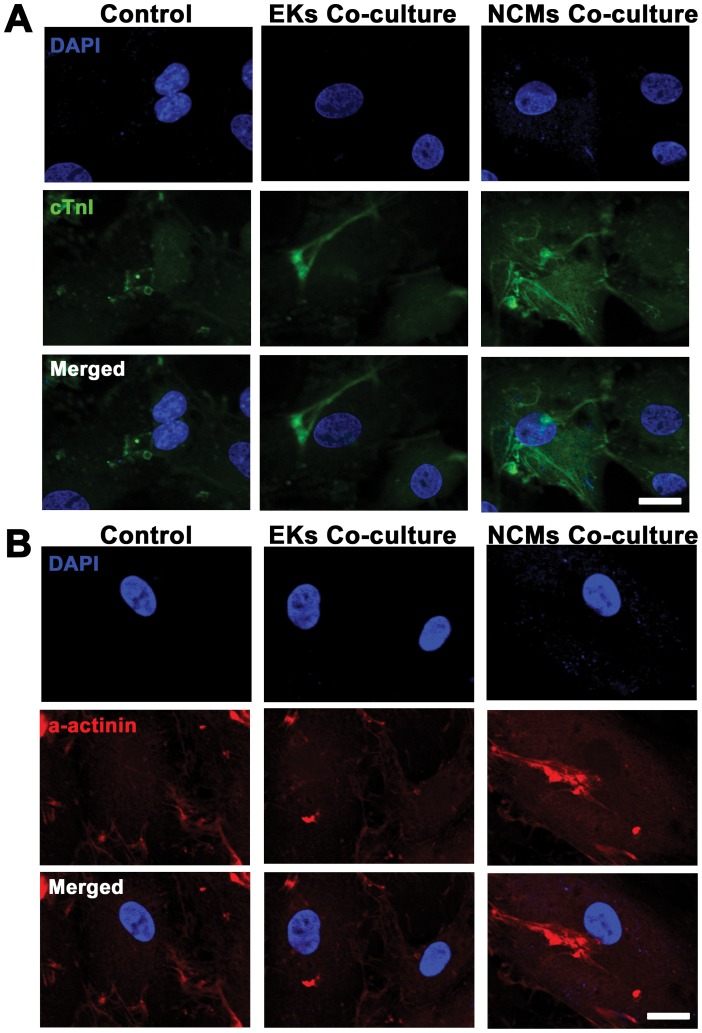
Immunostaining of cardiac specific proteins in ESCMs at day 20 of differentiation. **A,** Cells from beating outgrowths of EBs were incubated with primary antibody cTnI followed by FITC- conjugated secondary antibody (green). **B,** Cells from beating outgrowths of EBs were incubated with primary antibody α-actinin followed by Cy3-conjugated secondary antibody (red). Nuclei in the same field were stained with DAPI (blue). Merged figures were made by *FV10-ASW* Systems. Scale bars = 25 µm.

To further characterize the CMs derived from ESCs, immunostaining of cardiac troponin I (cTnI) and α-actinin was performed in the beating EB outgrowths to examine the cardiac specific proteins (Figure 4). Cardiac cTnI staining showed some unorganized myofilaments in EKs co-culture group and control group, while well-organized sarcomeric myofilaments in cytoplasmic patterns in NCMs co-culture groups. Immunostaining of α-actinin demonstrated the similar result that CMs derived from ESCs showed well-organized parallel striated patterns in NCMs co-culture group, but not in EKs co-culture group and control group. The morphology phenotype was similar to the highly organized, parallel bundles in cells from biopsies of heart. These data indicated that the cardiac specific proteins were present in differentiated EBs and the CM differentiation efficiency of ESCs was improved with NCMs co-culture.

### NCMs Co-culture Maintain the Function of the ESCMs

**Figure 5:**
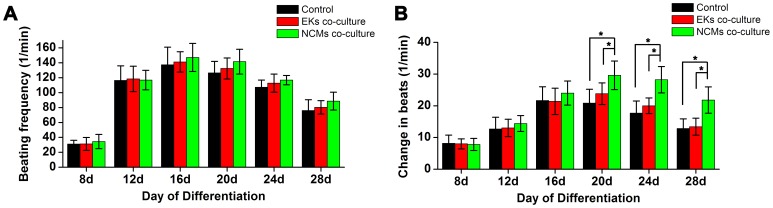
Effects of β-adrenergic stimulation on ESCMs. **A,** There are no baseline difference in beating frequency of ESCMs during plating culture. **B,** Changes in beating frequency after isoproterenol administration during the differentiation course. *: P<0.01.

There was no significant difference in the spontaneous beating frequency in the ESCMs of each group during the development of EBs (Figure 5A). We next sought to determine whether the beating EB outgrowths showed a change in contractile properties in response to the β-adrenergic agonist isoproterenol (Figure 5B). Indeed, a clear increase in beating frequencies were observed after washing in with 1 µmol/L isoproterenol, which resulted in almost 15∼30% increase in the beating frequencies. Co-culture with NCMs had no effect on the increase in beating frequency of ESCs-derived EBs before day 16 of differentiation (p>0.05), but had an effect on increasing the beating frequency after 20 days (p<0.01). Compared to EKs co-culture and control group, the beating frequency of EBs outgrowths with NCMs co-culture was significantly increased after 20 days (p<0.01). These results demonstrated that β-adrenergic receptors and the functional maintenance of positive inotropic response were more prominent when co-culture with NCMs.

### NCMs Co-culture Promote the Proliferation of ESC-derived CMs

**Figure 6:**
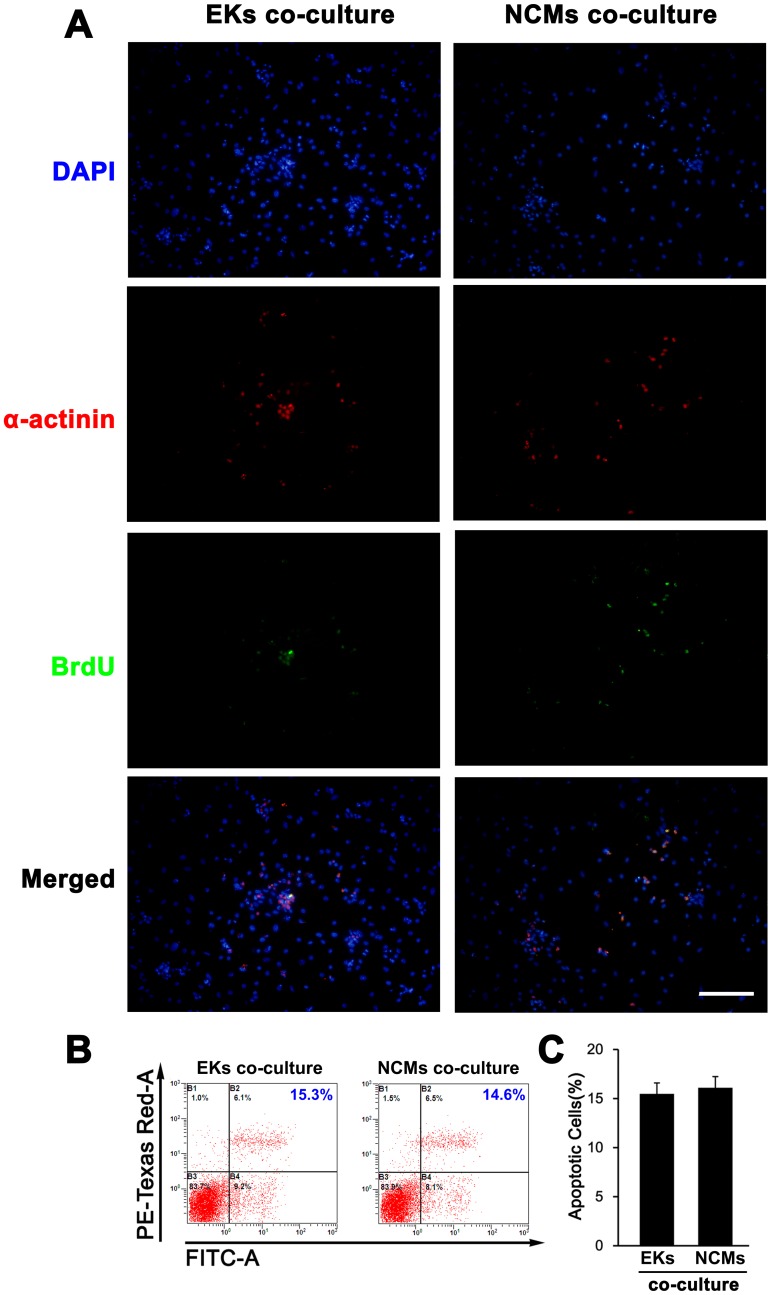
Cell proliferation and apoptosis assay of ESCs-derived CMs at day 20 in the co-culture conditions. **A,** Co-staining of BrdU and cardiac markers (a-actinin) to determine the effect of NCMs co-culture on the proliferation of ESCs-derived CMs. Note that the BrdU^+^ a-actinin^+^ cardiomyocytes were markedly increased with NCMs co-culture, compared with EKs co-culture (n = 5). The percentage of positive staining was calculated on the basis of the total number of cells in each view. **B,** Cell apoptosis assay by flow cytometry using Annexin V-FITC apoptosis assay kit at late-stage differentiation. **C,** Statistical analysis on flow cytometry results shows that there was no baseline difference in cell apoptosis in each group (n = 5). Scale bars = 100 µm.

To determined if the promoting effect of NCMs co-culture on CM differentiation of ESCs are duce to cell proliferation, co-staining of BrdU and α-actinin were performed to evaluate effect of NCMs co-culture on the proliferation of ESCMs in intermediate-stage (data not shown) and late-stage. In late stage (day 20), there are 9%±2% of BrdU^+^ α-actinin^+^ cells in EKs co-cultutre group, while 15%±4% of BrdU^+^ α-actinin^+^ cells in NCMs co-culture group (Figure 6A), suggesting that NCMs co-culture may promote the proliferation of ESC-derived CMs. We also considered the possibility that increased proliferation of ESC-derived CMs was due to reduction of cell apoptosis with NCMs co-culture. To address this possibility, we performed Annexin V-FITC apoptosis assay by flow cytometry, finding that there was no baseline difference in apoptosis of ESC-derived CMs in all groups (Figure 6B, C). These data indicated that NCMs co-culture may improve the CM differentiation efficiency of ESCs by promoting the proliferation of ESC-derived CMs in late-stage of differentiation.

## Discussion

Generating sufficient CMs that are pure and mature from ESCs remains challenging [21]. The potential use of ESCs to replace functional loss of particular tissues may depend on efficient differentiation protocols to derive tissue-specific cells. By manipulating the culture conditions in which ESCs differentiate, it has been possible to control and restrict the differentiation pathways and thereby generate cultures enriched in lineage-specific cells in vitro. Here, we develop a novel *in vitro* culture model to investigate cardiomyogenic differentiation and to enhance long-term functional maintenance of the ESCMs.

Co-cultures with cell culture inserts to study cells interactions during normal and special development, expansion, and differentiation have been used [22], [23], [24]. Co-culture with neonatal CMs has been reported as one of the microenvironment factors for transdifferentiation of mesenchymal stem cells into CMs [24]. Here, such a co-culture model has been established using ESCs, defined cells and Millicell™ cell culture inserts. We used the co-culture model instead of conditioned medium collected from cardiac cell cultures to treat the embryoid bodies (EBs). In the indirect co-culture model, two cell populations that are co-cultured in different compartments (insert and well) stay physically separated, but may communicate via paracrine signaling through the pores of the membrane.

Gene expression experiments in ESCMs revealed that the expression of cardiac markers, including GATA-4, Nkx2.5, ANF,α-MHC, MLC2a, and MLC2v, were augmented with NCMs co-culture. Nkx2.5 is an important marker genes used to confirm the CM differentiation in pluripotent stem cells [25]. The expression of Nkx2.5 indicates pluripotent stem cells preferentially differentiate into ventricular cells [26]. In the present study, Nkx2.5 appeared in the ascorbic acid-induced CM differentiation from ESCs, which is consistent with previous Takahashi et al.’s work [6]. GATA-4 is present in precardiac mesoderm and subsequently in the endocardial and myocardial layers of heart tube and developing heart [27]. Real time PCR analysis on the GATA-4 expression, detected as early as day 4 and continued throughout differentiation, is consistent with the known that GATA-4 transcription factor appears before the expression of other cardiac genes and is important in CM differentiation of ESCs. Atrial natriuretic factor (ANF) is considered to be a marker of chamber (atrial or ventricular) working myocardium [28]. MLC2v and MLC2a indicate that the differentiation toward ventricular or atrial phenotype is occurred. Other markers, such as MHC, are used to evaluate the cardiomyocyte maturation of differentiated embryonic stem cells. Real time PCR analysis on GATA-4, ANF and α-MHC showed that their expressions were relatively maintained by NCMs co-culture in prolonged culture time course. Compare with EKs co-culture, NCMs co-culture improves the efficiency of ESCs differentiation into CMs.

To identify long-term functional maintenance of ESCMs, we tested contractile properties by β-adrenergic agonist isoproterenol during CM differentiation of ESCs. We found that the increase in beating frequency was similar in both groups before 16 days, but became significantly different in the 20 days and more significantly different after 24 days. It is concluded that microenvironment created by co-culture with NCMs can influence differentiating efficiency and long-term maintain the CM differentiation from ESCs. In addition, BrdU immunostaining in late-stage cells revealed that the high proliferation was observed in those EBs in NCMs co-culture. These results suggested the effects of co-culture with NCMs might limit to the late-stage of differentiation, targeting proliferation of ESCMs.

This is the first time that neonatal CMs as a cellular source of signals that results in ESCs differentiating to CMs have been demonstrated. The co-culture model established here proved to be a useful tool for studying the paracrine interaction of different cell populations, investigating molecular mechanisms and signaling pathways leading to efficient differentiation, and studying the phenotypic control of derived cells after differentiation. Careful stepwise analysis on ESC differentiation and mimicking endogenous signals from NCMs are the most likely to increase and maintain the efficiencies of ESCs as well as other pluripotent stem cells differentiation into CM lineages.

## Materials and Methods

### Generation of αMHC-GFP Transgenic Mice

To generate αMHC-GFP transgenic mice, eGFP-Rex-Neomycin cDNA was sucloned into the expression vector containing a-myosin heavy chain promoter [29]. This Plasmid (αMHC-eGFP-Rex-Neo, No. 21229) was obtained from Addgene. Pronuclear microinjection and other procedures were performed by Cyagen Biosciences according to the standard protocols. Genotyping was performed by PCR on tail DNA with the following primer: eGFP forward: 5′-ACGTAAACGGCCACAAGTTC-3′; eGFP backward: 5′- GATCTTGAAGTTCACCTTGATGC-3′.

### Cell Culture of ESCs

Mouse CGR8 ESCs, previously established from strain 129P2/Ola mouse embryos by Smith et al. [30], were kindly provided by Prof. Duanqing Pei. Cells were cultured on 0.2% gelatin-coated plastic petri dishes without feeder cells in Dulbecco’s modified Eagle’s minimal essential medium (DMEM, Gibco, Invitrogen Corporation, Grand Island, NY, USA) supplemented with 15% fetal bovine serum (Gibco), 0.1 mmol/L nonessential amino acids (Sigma, St. Louis, MO, USA), 0.1 mmol/L β-mercaptoethanol, penicillin (100 U/mL), streptomycin (100 µg/mL), and 100 U/mL leukemia inhibitory factor (LIF) (Chemicon International Inc., Temecula, CA).

### Isolation and Culture of NCMs and EKs

NCMs were obtained from enzymatically isolated crude cellular fractions from neonate mouse ventricle as described previously [31]. Animal experiments were approved by the Fourth Military Medical University on the Use and Care of Animals. Myocyte isolation was conducted in accordance to Institutional Animal Care and Use Committee Guidelines. 1-day-old αMHC-GFP transgenic mice, identified by genotype PCR, were euthanized by injection of pentobarbital (80 mg/kg). The hearts were quickly excised, and washed with normal Tyrode solution. Ventricles were trimmed free of atria and major blood vessels, minced and placed in 0.1% collagenase (Sigma) solution. After 20 min enzyme digestions, the released cells were filtered, centrifuged and resuspended. Only cardiomyocytes, which expressed GFP, were sorted from the mixed cells by reporter-based fluorescence-activated cell sorting (FACS). The sorted NCMs were co-cultured with EBs in DMEM supplemented with 20% ES cell-qualified FBS(Gibco), 2 mM GlutaMAX (Invitrogen), 0.1 mM nonessential amino acid(Invitrogen) at a density of 2×10^4^ cells/cm^2^.

EKs were obtained from the skin of newborn (2–3-day old) mice. The detached epidermal sheets from newborn (2–3-day old) mice were cut roughly into 1-mm-diameter pieces, and shaken in a flask with 0.1% trypsin-EDTA solution for 6–8 min at 37°C. The suspension was then filtered through a mesh (74 m pore size) and centrifuged at 400 g for 5 min. EKs were obtained as sediment, which predominantly consisted of basal cells, intermingled with stratum spinosum cells. Keratinocytes were cultured in keratinocyte serum-free medium (Gibco) with 25 g/ml bovine pituitary extract (Gibco). These EKs were used for reconstruction culture after subculturing 2 or 3 times for 2 weeks. EKs were observed and photographed under a phase-contrast inverted microscopy (Olympus Optical Co. Ltd.) to evaluate their appearances.

### Indirect Co-culture Model

The 100 mm uncoated Petri dishes (Greiner Bio-One, Monroe, NC) were used to form EBs. After pipetting the 20 µL drops (400 cells) onto lids, lids were placed back on the 100 mm dish containing 10 mL PBS to prevent drying out of hanging drops. Each time there were 200 EBs formed in a 100 mm dish. On Day 3 of hanging drop culture, the cell aggregates were transferred and cultured in suspension in cell culture flasks (BD Bioscience) with differentiation medium for additional 2 days. 5-day-old EBs were carefully transferred to the 6- or 12-well plates coated with 0.2% gelatin and continuously cultured in differentiation medium. The medium for differentiation was above-mentioned ESC culture medium without LIF, but 0.1 mmol/L ascorbic acid (Sigma) was added to induce CM differentiation [6]. As previously described by Maltsev et al. [3], early-stage differentiation (shortly after the initiation of contractions) was day (8+3), intermediate-stage differentiation was day (8+8), and late-stage differentiation was day (8+16).

The Millicell™ hanging cell culture inserts (PET membranes with 1 µm pores) (Millipore, Bedford, MA, USA) can be especially used for co-culture. Two cell populations that are co-cultured in different compartments (insert and well) stay physically separated, but may communicate via paracrine signaling through the pores of the membrane. Here, 5-day-old EBs were transferred to 6- or 12- well plate coated with 0.2% gelatin, then the inserts were placed in some well of 6- or 12- well plate followed by EKs or the NCMs seeding on the inserts. Culture medium was the mentioned differentiation medium and changed every day. The PET membrane with 1 µm pores will become translucence in the presence of water, so we can observe the cells in the upper chamber or on bottom through a microscope. Spontaneously contracting cells appeared as clusters in outgrowths from the EBs. With daily gentle media changes and low EB density, the EBs continued to contract in culture for a period of observation of up to 36 days.

### Semi-quantitative Reverse Transcription-PCR

Semi-quantitative reverse transcription (RT)-PCR for MLC2v, MLC2a, α-MHC, ANF, Nkx2.5, GATA-4 and GAPDH was performed using standard procedures. Briefly, total RNA was prepared using Trizol reagent (Invitrogen). First strand cDNA was synthesized from 1 µg of total RNA, in a total volume of 20 µL, using oligo (dT)_18_ primer and a RevetAid™ First Strand cDNA Synthesis Kit. The RT-PCR was performed with GAPDH mRNA as a normalizing internal control. The resulting cDNA (50 ng) was amplified by PCR using specific primers. Primer sequences and PCR conditions are detailed in Table S1. Thermal cycling (in 20 µL) was performed as follows: a 3 min denaturation at 94°C, 30 cycles of 94°C for 30 sec, 60°C for 30 sec and 72°C for 1 min, and a final extension for 6 min at 72°C. PCR products were resolved by electrophoresis on 1.5% agarose gels. They were visualized by UV transillumination and photographed. Semi-quantitative analysis was done by Alphaview 1.3 software (Alpha Lnnotech Inc.).

### Real-Time PCR

For quantitative analysis on GATA-4, ANF, and α-MHC expressions, real-time PCR using above primers (detailed in Table S1) was performed as described previously [32]. Briefly, the processes of RNA extraction and reverse transcription of RNA (1 µg) were the same to Semi-quantitative RT-PCR. Real-time RT-PCR amplification reactions was performed in a final volume of 20 µL containing 50 ng cDNA, 10 µL of 2× iQSYBR-green mix (Takara, Japan), 300 nmol of forward and reverse primers using the LineGene 9660 real-time PCR Detection System (Bioer, China). The thermal cycling conditions comprised 95°C for 10 sec, 1 min at the corresponding annealing temperature, 53°C for 10 sec and 72°C for 40 sec. These settings were applied for 50 cycles. Specificity of amplification was determined by DNA melting curve during gradual temperature increments (0.5°C). The transcripts for GAPDH were used for internal normalization. Relative quantification was performed by the △△C_T_ method.

### Confocal Microscopy

EB outgrowths were fixed in 4% paraformaldehyde for 30 min, permeabilized for 15 min with 0.25% Triton X-100, and blocked in 5% normal goat serum (NGS) for 15 min. Subsequently, cells were incubated with the primary antibody in a humidified chamber at 37°C for 2 h. Rabbit anti-cardiac troponin I (cTnI) antibody (Santa Cruz, CA) and anti-α-actinin antibody (sigma) were added at dilutions of 1∶250 and 1∶400, respectively. After washed with 0.4% Triton X-100 and PBS, cells were incubated at 37°C for 4 h to corresponding FITC-conjugated or Cy3-conjugated secondary antibodies at a dilution of 1∶400. DAPI staining (Sigma, 1∶1000) was used to identify nuclei. Analysis was performed using a confocal microscope (FV1000, Olympus).

### β-Adrenergic Stimulation

The functional expression of ß-adrenergic receptors of ESCMs was tested as described previously [33]. The relative homogeneous beating EB outgrowths were chosen to study their response to adrenergic stimulation by isoproterenol. Contractions per minute of the EB outgrowths were measured under basal conditions and then after superfusion with 1 µmol/L isoproterenol. 10 sec after isoproterenol was added, we begin to assess the beating frequency for 5 min. The increase of beating frequencies in co-culture group was compared with control group.

### Cell Proliferation and Apoptosis Assay

Cell apoptosis was determined by flow cytometry using Annexin V-FITC apoptosis assay kit as previously reported [34]. Cells were stained with annexin-V and 7-amino-actinomycin D (7-AAD) for 15 minutes according to the manufacturer’s instructions (BD Pharmingen). Within 1 hour after staining, cells were analyzed by flow cytometry using CellQuest software (Becton Dickinson). For cell proliferation assay, the samples were pulsed with 5-bromodeoxyuridine (BrdU) at 10 µmol/L for 18 hours before co-staining for BrdU and α-actinin. Rabbit anti-BrdU antibody (Santa Cruz, CA) and mouse anti-α-actinin antibody (sigma) were added at dilutions of 1∶500 and 1∶400, respectively. After washed with 0.4% Triton X-100 and PBS, cells were incubated at 37°C for 2 h to corresponding FITC-conjugated or Cy3-conjugated secondary antibodies at a dilution of 1∶400. The cells were counterstained with DAPI (Sigma, 1∶1000) and analyzed using a fluorescence microscope. The experiments were repeated at least three times.

### Statistical Analysis

The ESC differentiation experiments were performed at least three times. All values are presented as mean±SEM. One-way ANOVA followed by Newman Keuls test was used for multiple comparisons. Differences with *p*<0.05 were considered statistically significant.

## Supporting Information

Table S1 **Primers and cycling conditions for RT-PCR.**
(DOC)
