# Supplementary material for: The Long-Term Differentiation of Embryonic Stem Cells into Cardiomyocytes: An Indirect Co-Culture Model
Source: PLoS One. 2013 Jan 28;8(1):e55233. doi: 10.1371/journal.pone.0055233 (PMC3557249; doi:10.1371/journal.pone.0055233)
Supplement: Table S1 — Primers and cycling conditions for RT-PCR. (DOC) [file pone.0055233.s001.doc]

**Table S1: Primers and cycling conditions for RT-PCR**

| Target Gene | GeneBank Number | Primer Sequence (5’-3’) | Size (bp) | Tm |
| --- | --- | --- | --- | --- |
| MLC2v | *NM_010861* | AAAGAGGCTCCAGGTCCAAT CCTCTCTGCTTGTGTGGTCA | 177 | 60 |
| MLC2a | *NM_022879* | TCAGCTGCATTGACCAGAAC AAGACGGTGAAGTTGATGGG | 148 | 60 |
| α-MHC | *NM_010856* | TGAAAACGGAAAGACGGTGA TCCTTGAGGTTGTACAGCACA | 132 | 60 |
| ANF | *NM_008725* | GGGGGTAGGATTGACAGGAT  AGCTGCGTGACACACCACAAG | 149 | 60 |
| Nkx2.5 | *NM_008700* | AAGTGCTCTCCTGCTTTCCCAG TTGTCCAGCTCCACTGCCTTC | 131 | 60 |
| GATA-4 | *NM_008092* | TCAAACCAGAAAACGGAAGC GTGGCATTGCTGGAGTTACC | 117 | 60 |
| GAPDH | *NM_008084* | TGTGTCCGTCGTGGATCTGA TTGCTGTTGAAGTCGCAGGAG | 150 | 60 |

**Abbreviations:** *MLC2v*, myosin light chain 2 ventricular transcripts; *MLC2a,* myosin light chain 2 atrial transcripts; *α-MHC*, α-myosin heavy chain; *ANF*, atrial natriuretic factor; *Nkx2.5*, NK2 transcription factor related, locus 5; *GATA-4*, GATA-binding protein 4; *GAPDH,* glyceraldehyde-3-phosphate dehydrogenase; *RT-PCR*, real-time PCR; *bp*, base pairs.
